# Reheating-Induced Matrix Reorganisation Modulates the In Vitro Digestibility of a Retrograded Normal/High-Amylose Maize Starch Blend

**DOI:** 10.3390/foods15183344

**Published:** 2026-09-21

**Authors:** Zeping Shao, Caili Li, Xiang Gao, Dan Li, Huan Lv, Shuo Wang

**Affiliations:** 1 Tianjin Key Laboratory of Food Science and Health, School of Medicine, Nankai University, Tianjin 300071, Chinaxianggao@mail.nankai.edu.cn (X.G.);; 2 Key Laboratory of Special Diet Nutrition and Health Research of China National Light Industry Council, School of Medicine, Nankai University, Tianjin 300350, China; 3 Centre for Nutrition and Food Sciences, Queensland Alliance for Agriculture and Food Innovation, The University of Queensland, Brisbane, QLD 4072, Australia

**Keywords:** high-amylose maize starch, starch blend, reheating, matrix architecture, in vitro hydrolysis

## Abstract

Reheating alters residual molecular order and matrix architecture in retrograded starch, but these changes do not necessarily produce parallel changes in enzymic digestion. Gels of normal maize starch (NM), high-amylose maize starch (HM), and a 50/50 (*w*/*w*) NM/HM blend were stored at 4 °C for 7 d and steam-reheated to core temperatures of 50 or 80 °C. HM was less hydrolysed than NM at either temperature. At 300 min, hydrolysis reached 51.3% and 61.4% for HM at 50 and 80 °C, respectively, versus approximately 81% for NM. DSC and XRD showed that HM-containing gels retained more thermally stable ordered structures. The 50/50 blend responded differently: reheating to 80 °C lowered hydrolysis from 72.6% to 65.6% despite no corresponding increase in residual order. This inverse response coincided with lower pore area (12.8 to 4.6 m^2^/g), larger 4 V/A pore diameter (966 to 2623 nm), and a more continuous freeze-dried morphology, consistent with fewer fine mercury-accessible pores and internal interfaces. Overall, starch structural composition established the broad ranking of hydrolysis after reheating, whereas matrix reorganisation modified the relationship between residual order and enzymic susceptibility in the 50/50 blend. Residual molecular order alone was therefore insufficient to predict the digestion response after reheating.

## 1. Introduction

Starch is the principal glycaemic carbohydrate in many diets, and its rate and extent of digestion influence glucose release from starch-containing foods. Retrogradation during cooling and storage promotes reassociation of gelatinised amylose and amylopectin chains and can generate type 3 resistant starch (RS3) and other structures with reduced enzymic susceptibility [1,2,3,4]. Foods containing retrograded starch are nevertheless often reheated before consumption. Reheating may partially melt double helices and crystallites, soften the matrix, and permit further molecular and matrix reorganisation. The extent to which retrogradation-derived enzyme resistance is retained after reheating therefore remains unclear.

Normal maize starch (NM) contains predominantly amylopectin, whereas high-amylose maize starch (HM) contains a greater proportion of long, relatively linear glucan chains [5,6,7,8]. During cooling, these chains participate in double-helical aggregation, B-type crystallisation, and dense non-crystalline associations that restrict enzymic hydrolysis [3,9,10]. High-amylose starch granules may also remain incompletely gelatinised under conventional hydrothermal processing. Granule-level organisation and crystalline polymorph can also influence the susceptibility of starch to amylolysis [11]. Consequently, the lower digestibility of processed HM can be attributed to contributions from residual granule architecture, structures formed during cooling, and local molecular packing, rather than amylose content alone. It remains uncertain whether starches differing in amylose content retain retrogradation-derived resistance to the same extent under an identical reheating treatment.

Reheating may affect these structural features differently across starch compositions. At temperatures within the melting range of retrograded structures, less stable ordered domains may be disrupted while more stable associations remain; increased chain mobility may also permit local rearrangement of the matrix. Most studies have focused on chain-length distribution, double-helical order, and crystallinity, although these measurements do not directly describe the physical accessibility of the polymer within the surrounding matrix. Enzyme action also depends on binding, transport, and local packing density, and intact or densely organised cereal and legume matrices can reduce starch hydrolysis even when crystalline order is limited [12,13,14,15,16,17,18]. A 50/50 NM/HM blend, therefore, provides a useful model in which molecular order and matrix organisation may not show intermediate or parallel responses.

In mixed starch systems, granules with different gelatinisation and swelling behaviours may also compete for available water during initial heating. Earlier-swelling granules can take up a greater share of water, potentially limiting the swelling of granules with higher gelatinisation resistance [19,20]. This effect may be particularly relevant to NM/HM blends, because NM and HM differ markedly in granule organisation, gelatinisation behaviour, and residual granule persistence after heating.

Previous work showed that microwave reheating altered residual order, texture, water distribution, and hydrolysis in retrograded normal maize starch gels [21]. The present study addressed a different question by comparing NM, HM, and a 50/50 (*w*/*w*) NM/HM blend under identical steam-reheating conditions. The target temperatures were chosen to bracket the gelatinisation transitions of the native starches and to represent contrasting reheating intensities. Gels were stored at 4 °C for 7 d and reheated to core temperatures of 50 or 80 °C. Debranched chain-length distribution, thermal properties, X-ray diffraction, freeze-dried morphology, mercury-accessible pore properties, texture, and comparative in vitro starch hydrolysis were evaluated. We hypothesised that HM-containing systems would retain greater structural resistance to hydrolysis after reheating, whereas matrix reorganisation in the blend could modify the relationship between residual order and enzymic susceptibility. The aim was to determine how starch composition and matrix-level organisation jointly shape the digestibility of reheated retrograded starch.

## 2. Materials and Methods

### 2.1. Materials

Normal maize starch (NM; apparent amylose fraction of 22.7% based on SEC analysis) and high-amylose maize starch (HM; 45.7%) were obtained from commercial sources (Quanyin Xiangyu, Biotechnology Co., Ltd., Beijing, China). All chemicals, including enzymes, were of analytical grade and sourced from Sigma-Aldrich (St. Louis, MO, USA) unless otherwise specified.

### 2.2. Starch Gel Preparation and Processing

Normal maize starch, HM, or a 50/50 (*w*/*w*) NM/HM blend was mixed with distilled water at a starch-to-water mass ratio of 1:2. The suspensions were heated in a water bath maintained at 90 °C for 20 min to prepare concentrated starch pastes. The pastes were transferred to cylindrical moulds, cooled at room temperature for 15 min, and stored at 4 °C for 7 d to allow retrogradation. The retrograded gels were then steam-reheated until their core temperatures reached either 50 or 80 °C, as monitored with a thermocouple probe. The target temperatures were selected with reference to the native-starch DSC transitions measured in this study: 50 °C was below the onset temperature of both NM and HM, whereas 80 °C was above the endset temperature of NM but within the broader transition range of HM. The two endpoints also represented mild and more extensive reheating conditions. Samples were immediately snap-frozen in liquid nitrogen after reheating, except those used for texture measurements, which were analysed fresh. Frozen samples were freeze-dried for subsequent structural analyses. Sample designations were as follows:

Native NM: native normal maize starch.

Native HM: native high-amylose maize starch (45.7% apparent amylose by SEC).

50/50: 50/50 (*w*/*w*) blend of NM and HM.

NM-retro, 50/50-retro, and HM-retro: retrograded gels without reheating.

NM-50, 50/50-50, and HM-50: retrograded gels were steam-reheated to a core temperature of 50 °C.

NM-80, 50/50-80, and HM-80: retrograded gels were steam-reheated to a core temperature of 80 °C.

### 2.3. Size-Exclusion Chromatography (SEC)

Starch molecular structure was characterised by SEC following established protocols. Samples (about 10 mg) were solubilised in 1.5 mL dimethyl sulfoxide (DMSO) containing 0.5% LiBr at 80 °C for 24 h. Insoluble residues were removed by centrifugation at 4000× *g* for 10 min, and the supernatant was precipitated with 10 mL absolute ethanol. The precipitate was recovered by centrifugation (4000× *g*, 10 min), washed with an additional 10 mL ethanol to eliminate residual DMSO/LiBr, and redispersed in 0.9 mL warm deionised water by heating in a boiling water bath for 10 min until fully dissolved. The dispersion was adjusted to pH 3.5 using acetate buffer and debranched with isoamylase at 37 °C for 24 h. The debranched mixture was snap-frozen in liquid nitrogen and lyophilised overnight. For SEC analysis, the dried debranched glucans were redissolved in DMSO/LiBr at 4 mg/mL by heating at 80 °C for 24 h, centrifuged at 4000× *g* for 10 min, and the supernatant was transferred to SEC vials for measurements [22,23,24]. The resulting distributions therefore represent the chain-length profiles recovered after solubilisation, debranching, and SEC analysis.

### 2.4. Differential Scanning Calorimetry (DSC)

Thermal properties were assessed using DSC (DSC 3, Mettler-Toledo, Greifensee, Switzerland). Freeze-dried samples (5 mg) were mixed with distilled water (1:3 *w*/*v*) in hermetic aluminium pans and equilibrated overnight at room temperature. Pans were heated from 20 °C to 110 °C at 10 °C/min, with an empty sealed pan used as the reference. Melting enthalpy (ΔH, J/g), onset (To), peak (Tp), and endset (Te) temperatures, as well as the temperature range (Te − To), were determined using STARe software (Mettler-Toledo, Greifensee, Switzerland). Measurements were performed in triplicate.

### 2.5. X-Ray Diffraction (XRD)

X-ray diffraction (XRD) patterns were obtained using a Bruker D8 Advance diffractometer (Bruker AXS GmbH, Karlsruhe, Germany) equipped with Cu Kα radiation. Ground and sieved samples were scanned over a 2θ range of 3–35° at a scan rate of 1° min^−1^ [9,21,23].

### 2.6. Scanning Electron Microscopy (SEM)

Freeze-dried gel fragments were mounted on aluminium stubs, sputter-coated with gold (10 nm thickness), and imaged using an SEM (ApreoC, Thermo Fisher Scientific Inc., Waltham, MA, USA) at 5 kV accelerating voltage. Micrographs were obtained at 1000× and 5000× to characterise the morphology of the freeze-dried starch matrices.

### 2.7. Mercury Intrusion Porosimetry (MIP)

Mercury intrusion porosimetry was used to characterise the mercury-accessible pore properties of the ground, freeze-dried samples using an AutoPore V 9620 instrument (Micromeritics, Norcross, GA, USA). Approximately 50–70 mg of sample, ground and passed through a 200-mesh sieve, was loaded into a 5-mL penetrometer. The samples were evacuated to <50 μmHg before mercury intrusion from 0.5 to 32,992 psia. Total intrusion volume, total pore area, volume- and area-based median pore entrance diameters, the 4 V/A diameter, bulk density, skeletal density, and porosity were obtained using MicroActive software (Micromeritics, Norcross, GA, USA) based on the Washburn model.

### 2.8. Texture Profile Analysis (TPA)

Fresh gels were subjected to two-cycle compression using a TA.newplus texture analyser (ISENSO, Shanghai, China). Samples were compressed to 30% of their original height. The test speed was 1 mm/s, with pre-test and post-test speeds of 1 and 5 mm/s, respectively. Hardness, springiness, cohesiveness, chewiness, gumminess, and adhesiveness were calculated from the force–time curves. Six measurements were conducted for each treatment [21].

### 2.9. In Vitro Digestion

Comparative in vitro starch hydrolysis was conducted under simulated gastric and intestinal conditions using a procedure adapted from the static INFOGEST method and our previously reported protocol [21,25,26]. Because the purpose was to resolve differences among the rapidly hydrolysed starch samples, the oral phase was omitted, and the pancreatin concentration was reduced to one-tenth of that used in the standard protocol.

A 40 mg portion of ground and sieved starch sample was weighed into a 50 mL centrifuge tube. The sample was subjected to gastric digestion in simulated gastric fluid (SGF) for 30 min at 37 °C with continuous agitation (200 rpm) in a shaking water bath. Thereafter, simulated intestinal fluid (SIF) containing pancreatin (Sigma-Aldrich, P1750) was added to initiate intestinal digestion (up to 6 h under the same conditions). For kinetic analysis, aliquots (50 μL) were withdrawn at designated time points (including a “0 h” sample taken after 30 min of gastric digestion but before pancreatin addition) and immediately mixed with 450 μL of 0.3 M Na_2_CO_3_ to terminate enzymatic activity. Following centrifugation (5000× *g*, 10 min), 100 μL of the supernatant was transferred to 1 mL of freshly prepared 4-hydroxybenzoic acid hydrazide (PAHBAH) reagent. The mixture was heated in a boiling water bath for 5 min, cooled, and absorbance was measured at 410 nm using a spectrophotometer. Reducing sugar content (expressed as glucose equivalents) was quantified against a standard curve to determine the extent of starch hydrolysis.

Preliminary experiments using the standard pancreatin concentration resulted in >90% hydrolysis within 15 min for all reheated samples. The reduced enzyme concentration was therefore used as a comparative analytical condition to resolve differences in hydrolysis kinetics. Accordingly, the results are interpreted as relative enzymic susceptibility under the present assay conditions rather than as standardised resistant starch contents.

Starch hydrolysis (%) = (glucose equivalents released × 0.9/initial dry starch mass) × 100

### 2.10. Statistical Analysis

Results are presented as mean ± standard deviation. Group means were compared by one-way analysis of variance followed by Tukey’s HSD test at *p* < 0.05 using SPSS Statistics 30. All experimental data are presented as mean ± SD for at least duplicate measurements, except for XRD and MIP, which were conducted as single measurements and interpreted descriptively to support comparisons of crystalline order and pore structure, respectively. Statistical analysis was not applied to these datasets.

## 3. Results

### 3.1. Chain Length Distribution of Debranched Starch

**Figure 1:**
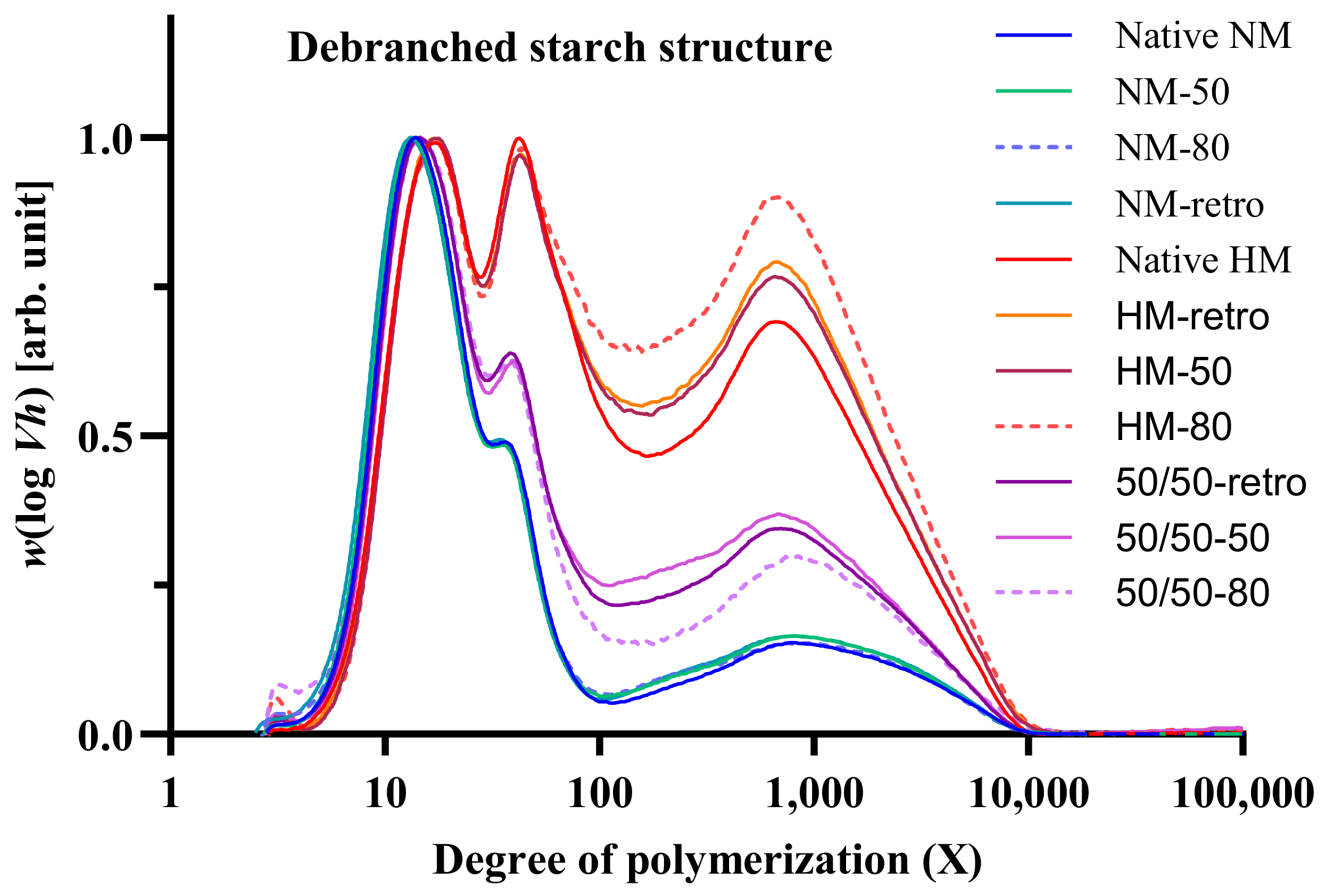
Chain length distributions of debranched starch samples. **Native NM**: native normal maize starch; **Native HM**: native high amylose maize starch with amylose content about 46%; 50/50: 50/50 (*w*/*w*) blend of normal maize starch and high-amylose maize starch; **NM-50**: The retrograded normal maize starch gel was reheated with steaming until the centre temperature reached 50 °C; **NM-80**: The retrograded normal maize starch gel was reheated with steaming until the centre temperature reached 80 °C. **Retro**: retrograded starch.

The chain length distributions (CLDs) of debranched starches are shown in Figure 1, expressed as weight distributions w(log Vh) normalised to the local maximum. Native NM was dominated by the AP1 region (DP < 36), with AP2 (DP 37–100) as a shoulder and an amylose fraction of 22.7%. Native HM showed separated AP1 and AP2 peaks, and a larger amylose fraction (45.7%), with a lower average amylose DP than NM (Table 1). The 50/50-retro sample had intermediate AM, AP1, and AP2 proportions. Retrogradation slightly shifted NM-retro’s amylose content upward without altering peak positions markedly, while 50/50-retro showed intermediate amylose proportions, with 50/50-retro blending the peak separations of both natives. Compared to normal maize starch, retrogradation and reheating exerted greater impacts on CLDs of high-amylose samples, especially altering the amylose peak.

Reheating induced subtle changes in CLDs (Table 1). For NM samples, reheating increased AP2 fractions, with minor reductions in amylose DP. In the 50/50 blend, reheating to 80 °C increased the AP1 proportion relative to 50/50-retro and 50/50-50, while the amylose and AP2 fractions decreased. HM-80 showed the highest apparent amylose proportion and AP2 average DP than HM-retro and HM-50. Overall, the changes were more apparent in the HM-containing systems than in NM.

### 3.2. Thermal Properties by DSC

**Figure 2:**
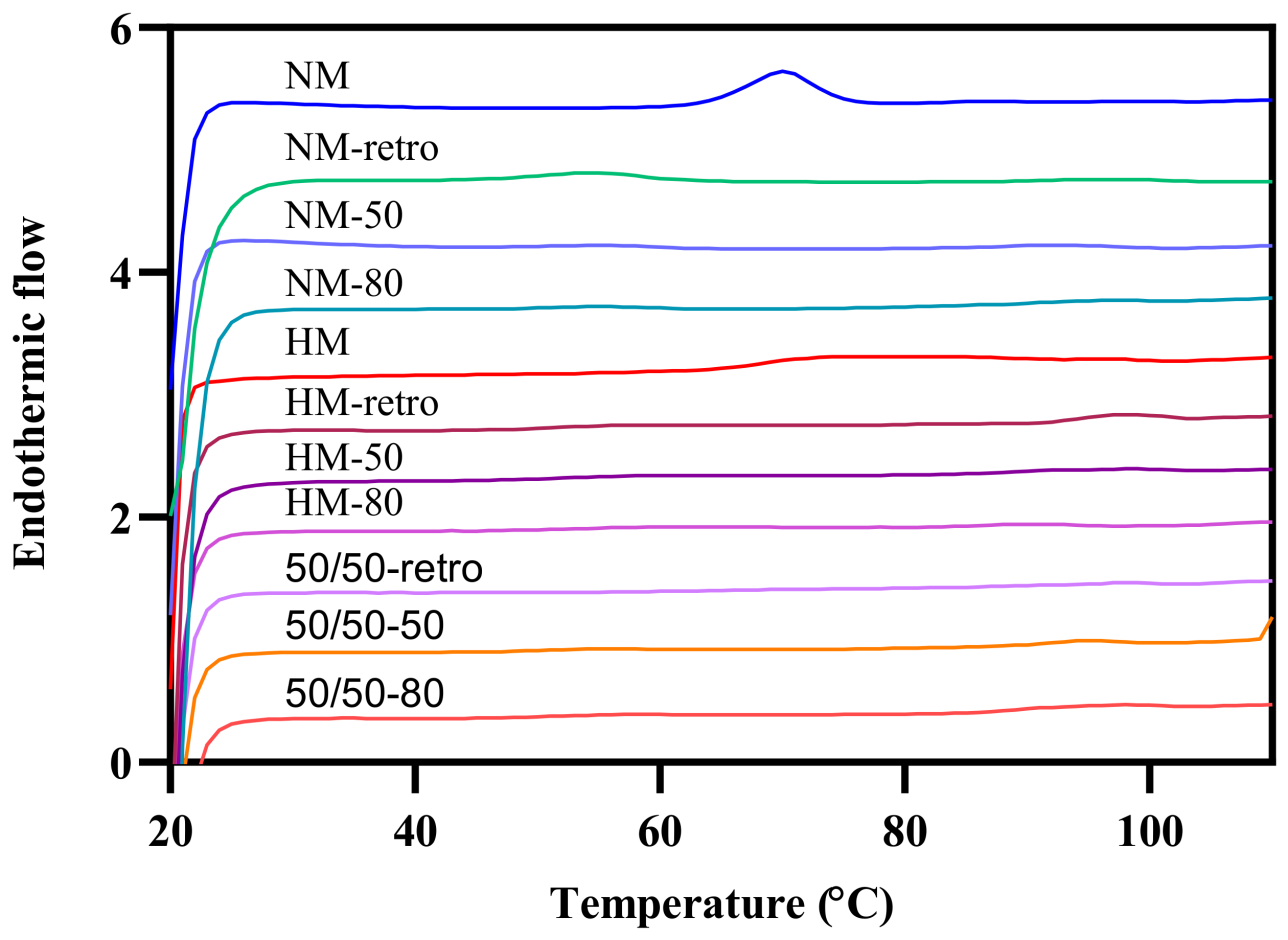
Differential scanning calorimetry profile of native starch, retrograded starch and reheating starch. **NM**: native normal maize starch; **HM**: native high amylose maize starch with amylose content about 46%; **50/50**: the mixture of 50% normal maize starch and 50% high amylose maize starch; **NM-50**: The retrograded normal maize starch gel was reheated with steaming until the centre temperature reached 50 °C; **NM-80**: The retrograded normal maize starch gel was reheated with steaming until the centre temperature reached 80 °C. **Retro**: retrograded starch.

The DSC thermograms of native, retrograded, and reheated starch gels are presented in Figure 2, with melting parameters summarised in Table 2. Native NM displayed a sharp endothermic peak with higher melting enthalpy (11.6 J/g) than Native HM (8.6 J/g. In contrast, Native HM exhibited a much broader transition extending to a higher endset temperature, consistent with the heterogeneous gelatinisation behaviour of high-amylose starch granules. After retrogradation, all samples showed low-enthalpy transitions at lower temperatures than the native starches. NM-retro showed a melting enthalpy of 3.6 J/g, whereas 50/50-retro and HM-retro showed lower values of 1.8 and 1.4 J/g, respectively. These transitions were consistent with the formation of less perfect recrystallised structures during storage.

Reheating impacted the detectable thermal stability differentially across starch types. Reheating to 50 °C preserved residual melting enthalpy in NM, 50/50, and HM samples. Reheating to 80 °C eliminated the detectable endotherm in NM. In contrast, the 50/50 blend and HM retained low but measurable enthalpy values of 0.6–0.8 J/g after reheating to 80 °C. The onset and peak temperatures of the 50/50 samples shifted slightly upward after reheating, although not all differences were statistically significant. This trend is consistent with the preferential melting of less stable or imperfectly ordered structures, leaving residual domains with greater apparent thermal stability.

### 3.3. Crystalline Structure Assessed by XRD

**Figure 3:**
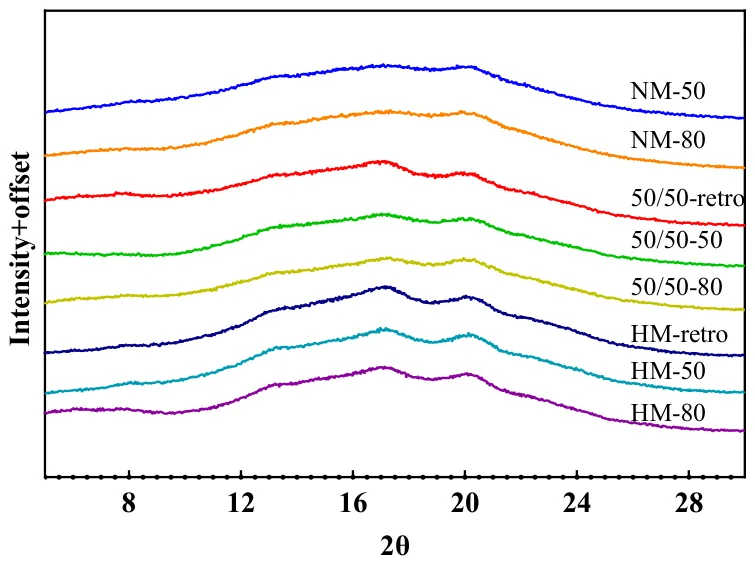
The X-ray diffraction diffractograms of the samples. **50/50**: the mixture of 50% normal maize starch and 50% high amylose maize starch; **NM-50**: The retrograded normal maize starch gel was reheated with steaming until the centre temperature reached 50 °C; **NM-80**: The retrograded normal maize starch gel was reheated with steaming until the centre temperature reached 80 °C. **Retro**: retrograded starch.

X-ray diffraction (XRD) patterns of the retrograded and reheated maize starch gels are shown in Figure 3. The diffraction patterns of the retrograded and reheated samples showed broad features at approximately 13°, 17°, and 20° 2θ, consistent with the presence of B- and V-type ordered structures [9,21,27]. The peak near 20° may reflect V-type single-helical order, including possible amylose–lipid complexes or retrograded amylose helices [21,27]. The absence of prominent native A-type diffraction features was consistent with extensive disruption of the original native-starch crystalline pattern during processing.

HM-retro showed the most pronounced diffraction features among the retrograded samples, while the 50/50 blend showed an intermediate pattern. Reheating broadened and reduced the apparent intensity of the diffraction features in NM, particularly after reheating to 80 °C. The NM-80 pattern was dominated by a broad amorphous halo, in agreement with the absence of a detectable DSC endotherm. In comparison, recognisable ordered features remained in the reheated HM-containing systems.

### 3.4. Morphology of Freeze-Dried Gels

**Figure 4:**
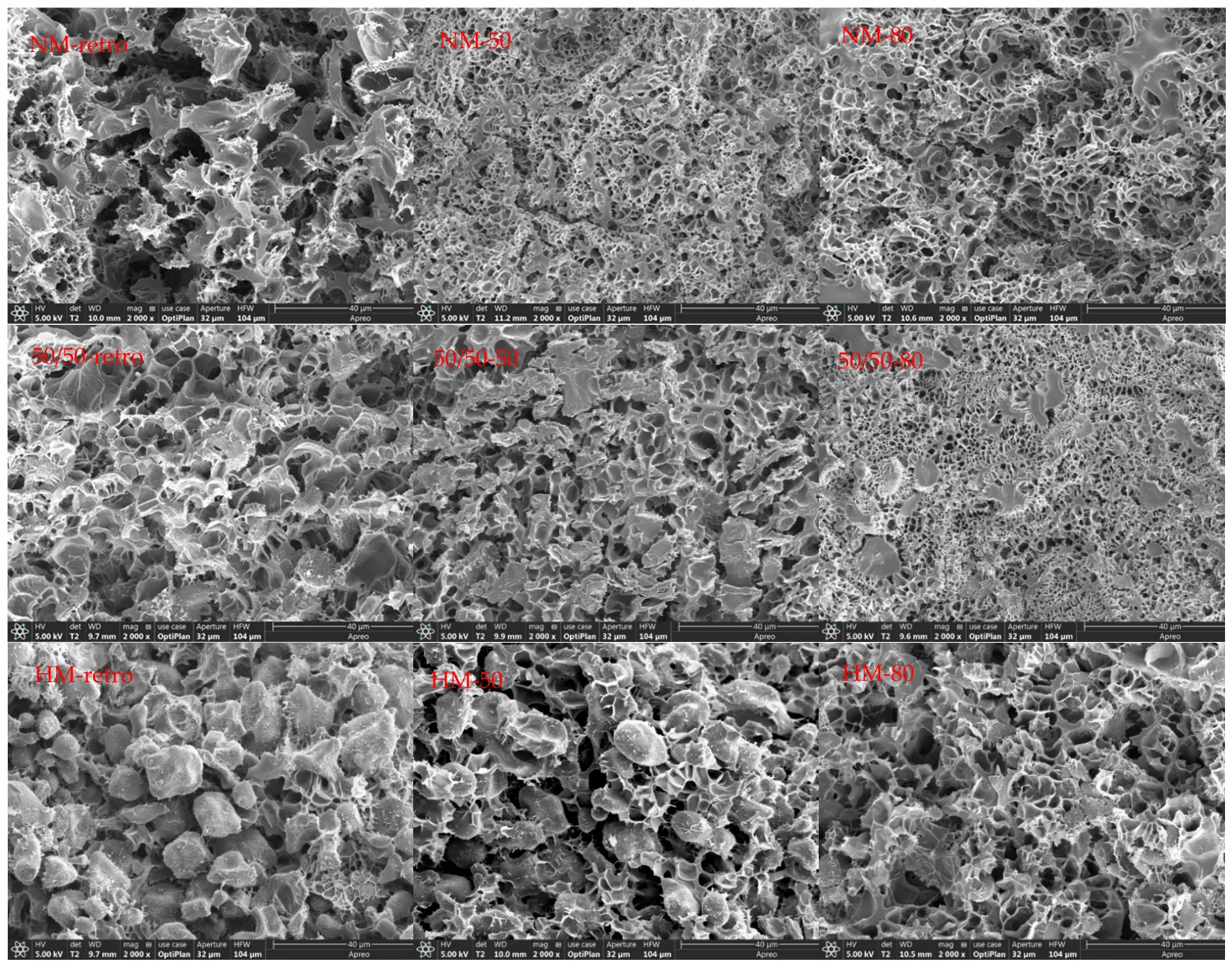
The Microstructure of starch gel and granules.

SEM micrographs (Figure 4) revealed distinct freeze-dried morphologies among the retrograded and reheated gels. Retrograded NM gels presented smooth and compact aggregates, whereas 50/50-retro showed a more heterogeneous matrix containing dense regions and residual granule-like structures. HM-retro contained more granule-like particles embedded within an irregular surrounding matrix.

Reheating altered these morphologies. NM-50 showed a relatively compact sponge-like network, whereas NM-80 showed a more open porous morphology. In the 50/50 system, 50/50-50 showed discontinuous domains separated by narrow fissures, while 50/50-80 appeared more continuous in the regions examined. HM-50 contained residual granule-like particles within a surrounding matrix, whereas HM-80 showed a more irregular porous morphology with fewer clearly defined granule-like structures.

### 3.5. Pore Structure by Mercury Intrusion Porosimetry

MIP was used to describe the mercury-accessible pore properties of the ground, freeze-dried samples. All samples exhibited high porosity (73.8–79.7%), with similar bulk density (0.3 g/mL) and skeletal density (1.2–1.3 g/mL) (Table 3). However, reheating temperature affected mercury-accessible pore characteristics differently among starch compositions.

In NM, reheating from 50 to 80 °C increased total pore area from 5.6 to 21.4 m^2^/g and decreased the 4 V/A diameter from 2184 to 513 nm. HM showed a similar trend, with pore area increasing from 2.1 to 9.4 m^2^/g and the 4 V/A diameter decreasing from 4402 to 1279 nm. In contrast, the 50/50 blend showed the opposite response: total pore area decreased from 12.8 to 4.6 m^2^/g, while the 4 V/A diameter increased from 966 to 2623 nm. Because the total intrusion volume remained similar, these changes are consistent with a reduced contribution from fine mercury-accessible pores and internal interfaces in 50/50-80 rather than an overall loss of pore volume.

### 3.6. Textural Properties by TPA

Textural parameters of retrograded and reheated gels are detailed in Table 4. Retrograded gels from the 50/50 blend and HM displayed markedly higher hardness than NM, with 50/50-retro and HM-retro reaching approximately 142.6 and 145.9 N, respectively. HM-retro, however, showed lower cohesiveness (0.54), indicating that HM-retro differed from 50/50-retro not only in firmness but also in internal structural continuity, whereas 50/50-retro balanced hardness with higher cohesiveness (0.80), suggesting a different balance between firmness and cohesiveness in the blend. The results were consistent with the microstructure observed by SEM, which showed that the HM sample had more intact starch particles, resulting in poor continuity of the starch gel. The texture data of NM-retro were summarised in a previous paper [21].

Reheating softened all gels, with hardness decreasing more strongly at 80 °C than at 50 °C. NM-50 and NM-80 exhibited the softest textures (~11.9 and 8.0 N, respectively), with NM-80 showing significantly lower chewiness and gumminess, reflecting extensive disruption of weak retrograded bonds. The 50/50-50 maintained intermediate hardness, significantly higher than NM-50 but lower than HM-50, accompanied by reduced springiness and cohesiveness. HM-50 and HM-80 showed the greatest hardness post-reheating (~28.4 and 16.4 N, respectively), yet with the lowest springiness at around 0.4. For reheated normal and 50/50 blend samples, adhesiveness decreased with the rise in heating temperature from 50 °C to 80 °C. Conversely, the adhesiveness of HM slightly increased. Overall, the texture results showed that HM-containing gels retained greater macroscopic firmness after reheating than NM, while the 50/50 blend showed an intermediate response.

### 3.7. In Vitro Digestion

**Figure 5:**
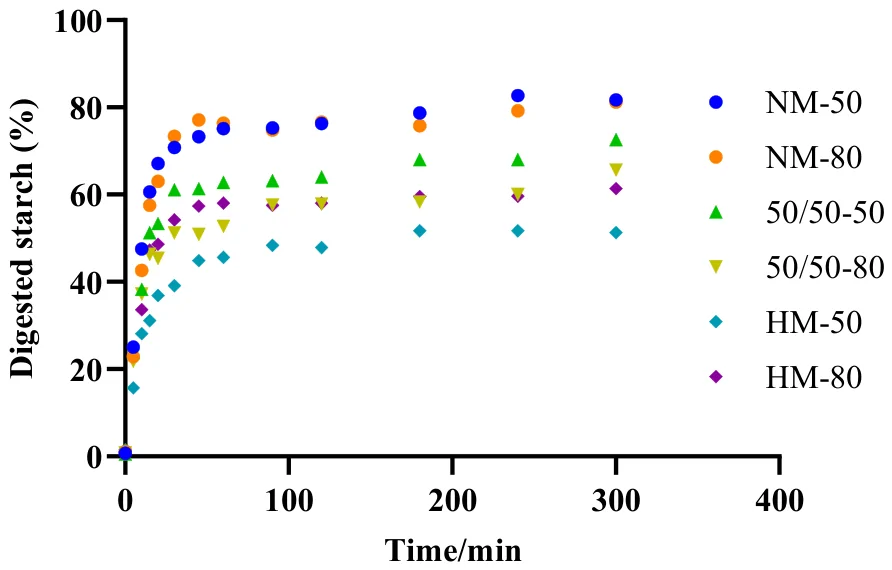
The in vitro enzymic hydrolysis of reheated retrograded starch gels.

The in vitro digestion profiles of reheated retrograded starch gels, assessed using a simulated gastrointestinal digestion model, revealed distinct hydrolysis kinetics influenced by starch type and reheating temperature (Table S1, Figure 5). All samples showed rapid early hydrolysis followed by a slower increase towards 300 min.

For NM, NM-50 and NM-80 exhibited comparable final hydrolysis, reaching approximately 81% at 300 min. NM-50 displayed faster initial hydrolysis with 67.1% digested starch at 20 min, compared to 63.0% for NM-80. In contrast, NM-80 showed a slightly higher hydrolysis at 60 min (76.4% vs. 75.1% for NM-50), the difference between the two samples became small at later digestion times.

The 50/50 blend presented intermediate digestion behaviours, with an overall lower hydrolysis degree than NM. Notably, at 300 min, hydrolysis was significantly lower in 50/50-80 than in 50/50-50 (65.6% vs. 72.6%). Initial hydrolysis was also faster in 50/50-50, with 53.4% digested starch at 20 min, compared to 45.4% for 50/50-80.

High-amylose (HM-50, HM-80) samples showed the lowest overall digestibility among the reheated samples. HM-80 was hydrolysed to a greater extent than HM-50 at 300 min (61.4% vs. 51.3%). These values were approximately 20% and 30% lower, respectively, than those of normal maize starch (~81%). Thus, HM retained greater resistance to enzymic hydrolysis after reheating, although its response to reheating temperature differed from that of the 50/50 blend.

## 4. Discussion

### 4.1. Starch Composition Establishes the Broad Digestibility Response After Reheating

Starch structural composition established the broad ranking of in vitro digestibility after reheating. The two endpoints imposed different thermal challenges: 50 °C was below the native gelatinisation onset of both starches, whereas 80 °C exceeded the native NM endset temperature but remained within the broader HM transition. This distinction helps explain why NM, HM, and the blend did not respond similarly under identical steam-reheating conditions. HM and the 50/50 blend retained more thermally stable ordered structures than NM, consistent with the greater contribution of long, relatively linear glucan chains to double-helical order, intermolecular association, and dense chain packing [3,5,6,9,28,29]. After reheating to 80 °C, NM showed no detectable retrogradation endotherm and only weak residual diffraction features, whereas low residual endothermic transitions and ordered diffraction features remained in the HM-containing samples. Furthermore, reheating preferentially disrupted the less stable ordered regions, short or defective ordered structures, leaving residual ordered structures with greater thermal stability, as indicated by the numerical upward shift in To (Table 2) [2,30,31]. SEM also showed more residual granule-like structures in the HM-containing samples, while texture analysis showed greater firmness than in NM. Long linear chains may contribute to both double-helical and crystalline order and dense amorphous packing, thereby reducing enzyme accessibility [3,10,12]. Taken together, the retention of residual granule architecture, thermally stable ordered structures, and dense chain packing was consistent with the lower hydrolysis of HM and the intermediate response of the 50/50 blend.

The chain-length distributions further showed that the response to reheating differed among the three starch systems. Only minor variations were observed within the NM series, indicating that its chain-length distribution remained stable after reheating. A more distinct shift occurred in the 50/50 blend. Compared with 50/50-50, 50/50-80 showed a higher AP1 proportion and a lower AP2 average DP, accompanied by a higher average DP of the AM fraction. These opposite shifts in the amylose and amylopectin regions do not indicate overall chain shortening or preferential cleavage of longer chains, but rather a redistribution of recovered chain populations after solubilisation and debranching [22,24]. The shift towards shorter AP2 chains was consistent with its lower residual crystalline order of 50/50-80, whereas the longer recovered AM chains may contribute to denser amorphous packing and provide a molecular basis for its more continuous post-reheating matrix. The different contributions of amylopectin chains to crystalline order and amylose chains to amorphous packing, therefore, help explain why residual order and enzymatic hydrolysis did not change in parallel in the 50/50 blend.

In HM, the proportions and average DPs of the AM, AP1, and AP2 fractions did not differ significantly among HM-retro, HM-50, and HM-80. The apparent AM proportion and AP2 average DP were numerically higher in HM-80, but these differences were not statistically significant. Importantly, HM-containing gels retained residual granules, and small SEC subsamples may contain different proportions of gelatinised matrix and granule remnants, which may result in differences in the apparent AM/AP in HM-containing samples. The higher hydrolysis of HM-80 than HM-50 was more likely related to partial disruption and reorganisation of the residual starch structure than to changes in chain length, consistent with reports that high-amylose starch digestibility is strongly affected by granule organisation, chain mobility, and molecular packing [6,9,10]. Overall, SEC described the chain-length distribution, whereas DSC and XRD characterised the residual ordered structures, and SEM and texture analysis provided complementary information at larger structural scales after reheating.

### 4.2. Matrix Reorganisation Contributes to the Lower Hydrolysis of the 50/50 Blend After Reheating to 80 °C

For the 50/50 blend, reheating from 50 to 80 °C altered the chain-length distribution, reduced residual order, and softened the gel, yet hydrolysis at 300 min decreased from 72.6% to 65.6%. This inverse response was also distinct from those of the individual starches and suggests that residual order alone could not explain hydrolysis and that matrix organisation was also important. This is consistent with evidence from processed high-amylose maize starches that enzyme resistance can arise from the combined effects of molecular and mesoscopic structural features rather than from a single measure of structural order [32]. Starch hydrolysis depends not only on the susceptibility of the substrate to enzymic attack, but also on whether enzymes can access and bind to the substrate.

SEM showed discontinuous domains separated by fine fissures in 50/50-50, whereas the regions examined in 50/50-80 appeared more uniform and continuous. Reheating-induced changes in molecular order, texture, and freeze-dried morphology have also been reported for retrograded maize starch gels [21,27]. In the present blend, the morphological difference was consistent with the lower hydrolysis at 80 °C.

The MIP results supported this morphological change. From 50/50-50 to 50/50-80, total pore area decreased from 12.8 to 4.6 m^2^/g, while total intrusion volume remained close to 3.0 mL/g. Meanwhile, the 4 V/A diameter increased from 966 to 2623 nm. The combination of similar intrusion volume, lower pore area, and a larger characteristic diameter indicates that fine pores and internal interfaces were reduced rather than there being an overall loss of pore volume. This agrees with the SEM observation of a more continuous matrix.

A possible origin of the blend-specific matrix is unequal water uptake during the initial gel preparation, followed by partial water redistribution during reheating. In a mixed population of NM and HM granules, NM granules would be expected to gelatinise and swell more readily, taking up a greater proportion of the available water during heating. This could leave less water available for HM granule swelling, limiting HM gelatinisation and producing a mixed matrix that differs from either NM or HM alone. Under this interpretation, the higher pore area of 50/50-50 may reflect a discontinuous matrix formed by preferential swelling of the NM fraction together with residual HM-rich domains. Reheating to 80 °C may have allowed partial water redistribution of these domains, forming a more continuous matrix with fewer fine internal interfaces.

**Figure 6:**
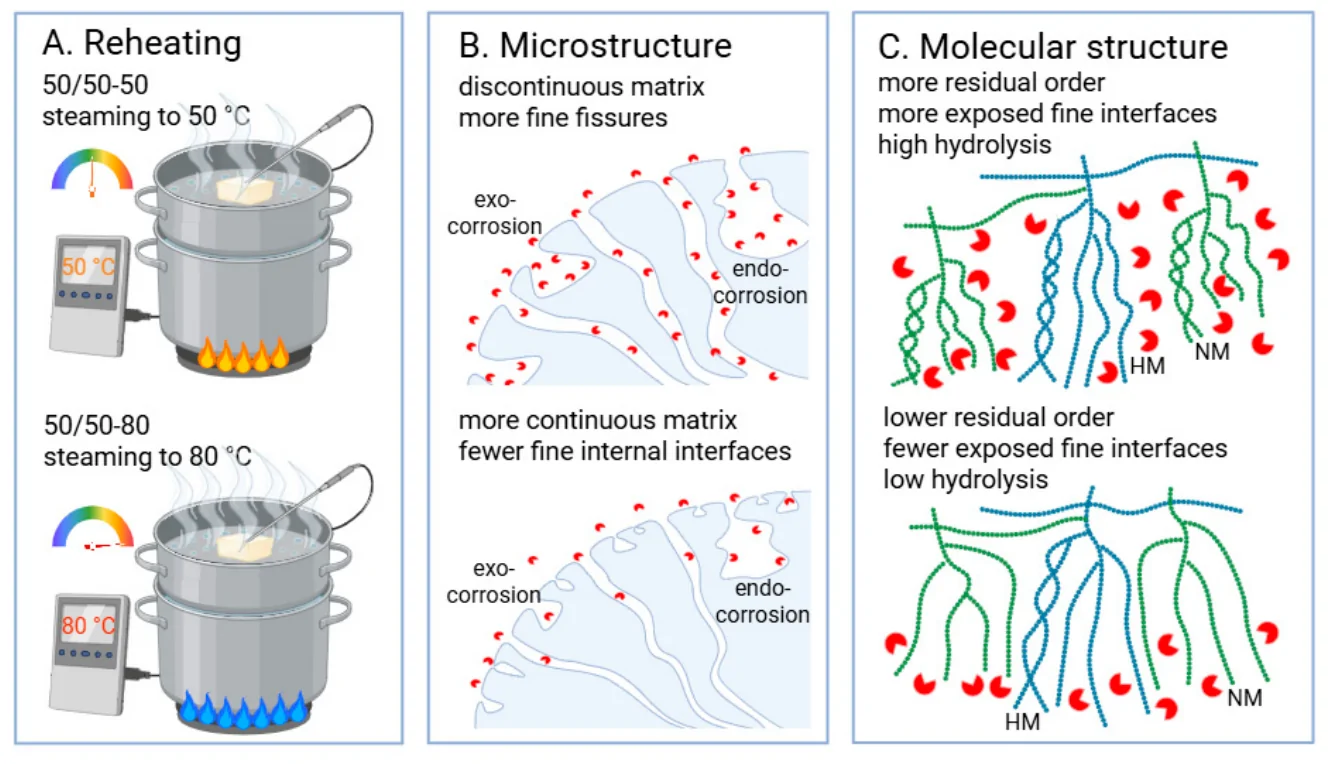
Proposed structural explanation for the lower in vitro hydrolysis of the 50/50 NM/HM blend after reheating to 80 °C compared with 50 °C. NM, normal maize starch; HM, high-amylose maize starch.

This reduction in fine surface area may correspond to less interfacial area available for enzyme attachment and could therefore have contributed to the lower hydrolysis of 50/50-80. In this system, the reduction in residual order was expected to favour hydrolysis, but the loss of fine internal interfaces and the formation of a more continuous matrix may have partly offset this effect. Thus, the more continuous matrix in 50/50-80 may have limited starch accessibility sufficiently to maintain lower hydrolysis despite lower residual order. Similar relationships between dense matrix organisation and reduced enzymic hydrolysis have been found in high-amylose wheat bread and tortillas [16,17,18]. This proposed mechanism is illustrated in Figure 6.

## 5. Conclusions

Starch structural composition established the broad difference in hydrolysis among the three starch systems. HM-containing samples retained more heat-stable ordered structures, firmer matrices, and more residual granule-like material, and were less hydrolysed than NM. The 50/50 blend showed an additional matrix-level response to reheating. Although heating to 80 °C reduced its molecular and crystalline order, hydrolysis fell from 72.6% to 65.6%. SEC, DSC, XRD, texture, SEM, and MIP together indicated that reheating to 80 °C was consistent with a more continuous structure with fewer fine accessible interfaces. Overall, residual molecular order alone did not predict the digestion response after reheating; instead, hydrolysis was associated with differences in starch molecular organisation, residual granule architecture, and matrix morphology. For dietary starch systems, formulation and reheating history should be considered jointly, although the implications for human glycaemic responses require direct validation.

## Figures and Tables

**Table 1:** SEC data from debranched starch.

Debranched Parameters									
Samples	AM (%)	AP1 (%)	AP2 (%)	AM Average DP	AP1 Average DP	AP2 Average DP	X_AM_	X_AP1_	X_AP2_
Native NM	22.7 ± 0.9 a	63.5 ± 0.8 e	13.8 ± 0.1 a	1497.8 ± 13.6 c	16.4 ± 0.1 abc	53.3 ± 0.1 a	802.2 ± 2.5 cd	36.6 ± 0.1 ab	13.9 ± 0.0 a
NM-retro	24.2 ± 0.1 a	63.2 ± 0.6 e	12.6 ± 0.5 a	1457.4 ± 5.5 bc	15.9 ± 0.0 a	53.7 ± 0.7 a	805.8 ±12.6 d	36.0 ± 0.9 a	13.1 ± 0.0 a
NM-50	24.2 ± 1.0 a	62.5 ± 1.3 e	13.2 ± 0.2 a	1451.5 ± 16.1 bc	16.2 ± 0.1 ab	54.2 ± 0.6 a	790.0 ± 9.8 cd	38.6 ± 1.4 bc	13.6 ± 0.0 a
NM-80	23.3 ± 0.8 a	63.0 ± 1.8 e	13.6 ± 0.9 a	1429.2 ± 18.9 bc	16.8 ± 0.1 bc	54.4 ± 0.9 a	840.9 ± 37.0 d	36.0 ± 0.1 a	13.6 ± 0.0 a
Native HM	45.7 ± 0.4 c	31.1 ± 0.4 b	23.2 ± 0.1 c	1161.7 ± 3.2 a	18.4 ± 0.0 d	64.7 ± 0.2 d	666.2 ± 22.0 a	42.0 ± 0.1 de	16.9 ± 0.1 b
50/50-retro	35.8 ± 1.1 b	46.2 ± 0.9 cd	18.0 ± 0.2 b	1235.7 ± 15.2 a	17.0 ± 0.1 c	61.1 ± 0.2 bc	743.1 ± 4.6 bc	38.5 ± 0.5 abc	14.5 ± 0.1 a
50/50-50	37.9 ± 2.9 b	44.3 ± 1.2 c	17.9 ± 1.7 b	1186.7 ± 24.1 a	17.1 ± 0.3 c	62.4 ± 0.6 c	689.1 ± 2.1 ab	40.3 ± 0.2 cd	14.5 ± 0.0 a
50/50-80	32.9 ± 1.3 b	49.8 ± 1.6 d	17.4 ± 0.4 b	1367.8 ± 46.1 b	16.9 ± 0.3 bc	59.6 ± 0.8 b	838.5 ± 7.9 d	38.4 ± 0.2 abc	14.6 ± 0.2 a
HM-retro	49.8 ± 0.0 cd	28.3 ± 0.5 ab	21.9 ± 0.5 c	1181.01 ± 32.3 a	18.4 ± 0.2 d	65.9 ± 0.3 de	670.4 ± 16.2 a	43.3 ± 0.4 e	16.9 ± 0.5 b
HM-50	49.5 ± 0.1 cd	28.5 ± 0.1 ab	22.1 ± 0.2 c	1188.2 ± 21.1 a	18.6 ± 0.1 d	65.8 ± 0.1 de	666.1 ± 14.0 a	43.0 ± 1.0 e	17.3 ± 0.4 b
HM-80	52.8 ± 2.7 d	25.7 ± 2.6 a	21.4 ± 0.1 c	1188.5 ± 48.7 a	18.5 ± 0.4 d	67.0 ± 0.4 e	687.7 ± 8.3 ab	43.3 ± 0.5 e	16.8 ± 1.2 b

AM: amylose content. AP1: the content of amylopectin with DP 0–36; AP2: the content of amylopectin with DP 37–100; X_AM_: the DP at the peak of AM; X_AP1_: the DP at the peak of AP1; X_AP2_: the DP at the peak of AP2. Values are mean ± SD from duplicate SEC measurements (*n* = 2). Values with different letters in the same column are significantly different with *p* < 0.05.

**Table 2:** The melting parameters of reheated starch measured by DSC.

	ΔH (J/g)	To (°C)	Tp (°C)	Te (°C)	Te − To (°C)
Native NM	11.6 ± 0.5	63.7 ± 0.3	69.9 ± 0.5	75.0 ± 0.6	11.2 ± 0.4
NM-retro	3.6 ± 0.2 d	45.7 ± 0.2 a	53.6 ± 0.4 a	60.5 ± 0.7 a	14.8 ± 0.6 a
NM-50	1.2 ± 0.2 abc	47.7 ± 0.7 ab	54.9 ± 0.8 ab	62.3 ± 0.2 abc	14.6 ± 0.5 a
NM-80	ND	ND	ND	ND	ND
50/50-retro	1.8 ± 0.4 c	45.7 ± 1.0 a	53.0 ± 0.7 a	60.6 ± 1.3 a	14.9 ± 0.3 a
50/50-50	0.8 ± 0.1 ab	48.6 ± 0.5 ab	54.4 ± 0.2 a	61.0 ± 0.2 ab	12.4 ± 0.3 a
50/50-80	0.6 ± 0.0 a	48.8 ± 1.0 ab	56.7 ± 0.0 ab	62.4 ± 0.4 abc	13.6 ± 1.4 a
Native HM	8.6 ± 0.7	64.9 ± 0.0	74.0 ± 0.4	95.2 ± 2.9	30.3 ± 2.9
HM-retro	1.4 ± 0.1 bc	50.7 ± 3.2 ab	56.4 ± 0.8 ab	66.8 ± 2.9 c	16.1 ± 0.3 a
HM-50	0.8 ± 0.1 ab	52.7 ± 2.8 b	58.7 ± 2.2 b	63.3 ± 2.8 abc	10.6 ± 5.6 a
HM-80	0.8 ± 0.0 ab	51.9 ± 1.0 b	58.7 ± 2.1 b	66.5 ± 0.6 bc	14.6 ± 1.6 a

Values are mean ± SD from at least duplicate measurements. Values with different letters in the same column are significantly different with *p* < 0.05. Native NM and Native HM were not included in the statistical analysis and are shown for reference. Native NM and NM-retro data were cited from our previous study [21]. ND, not detected.

**Table 3:** Mercury intrusion porosimetry parameters of reheated samples.

Parameter	NM-50	NM-80	50/50-50	50/50-80	HM-50	HM-80
Total intrusion volume (mL/g):	3.1	2.7	3.1	3.0	2.3	3.0
Total pore area (m^2^/g):	5.6	21.4	12.8	4.6	2.1	9.4
Volume-based median pore entrance diameter (nm):	12,167.8	17,193.9	18,152.1	7996.1	7930.3	11,784.6
Area-based median pore entrance diameter (nm):	420.2	64.1	84.8	711.0	2410.4	81.3
Average pore diameter (4 V/A) (nm):	2184.4	512.8	965.9	2623.2	4402.2	1278.6
Bulk density (g/mL):	0.3	0.3	0.3	0.3	0.3	0.3
Skeletal density (g/mL):	1.3	1.3	1.2	1.2	1.2	1.2
Porosity (%):	79.7	78.1	79.2	78.7	73.8	78.4

**Table 4:** The textural properties of retrograded and reheated starch gels.

	Hardness	Springiness	Cohesiveness	Chewiness	Gumminess	Adhesiveness
NM-50	11.91 ± 0.65 b	0.75 ± 0.05 c	0.66 ± 0.05 cd	5.94 ± 1.10 c	7.87 ± 1.02 ab	−0.11 ± 0.02 ab
NM-80	7.99 ± 0.88 a	0.69 ± 0.01 bc	0.71 ± 0.03 d	3.92 ± 0.50 ab	5.68 ± 0.65 a	−0.05 ± 0.03 a
50/50-50	19.57 ± 1.28 c	0.60 ± 0.04 b	0.44 ± 0.01 b	5.13 ± 0.68 bc	8.53 ± 0.81 b	−0.35 ± 0.15 b
50/50-80	10.92 ± 1.71 ab	0.61 ± 0.04 b	0.58 ± 0.04 c	3.87 ± 0.86 ab	6.31 ± 1.29 ab	−0.16 ± 0.09 ab
HM-50	28.24 ± 2.10 d	0.38 ± 0.03 a	0.27 ± 0.01 a	2.87 ± 0.53 a	7.60 ± 0.89 ab	−0.15 ± 0.03 ab
HM-80	16.36 ± 1.09 c	0.40 ± 0.01 a	0.35 ± 0.05 ab	2.28 ± 0.47 a	5.75 ± 1.20 a	−0.17 ± 0.21 ab
50/50-retro	142.58 ± 14.29	0.97 ± 0.04	0.80 ± 0.02	110.10 ± 3.00	113.84 ± 8.28	0.00 ± 0.00
HM-retro	145.94 ± 4.68	0.97 ± 0.04	0.54 ± 0.06	76.99 ± 13.63	78.99 ± 10.80	0.00 ± 0.00

Mean ± SD calculated from duplicate measurements. The values of 50/50-retro and HM-retro have not undergone data analysis and are for reference only. Values with different letters in the same row are significantly different with *p* < 0.05.

## Data Availability

The data presented in this study are available from the corresponding author upon reasonable request. The data are not publicly available due to privacy or ethical restrictions.

## Supplementary Materials

The following supporting information can be downloaded at: https://www.mdpi.com/article/10.3390/foods15183344/s1, Table S1: Digestion data of various samples.
