# Supplementary material for: Reheating-Induced Matrix Reorganisation Modulates the In Vitro Digestibility of a Retrograded Normal/High-Amylose Maize Starch Blend
Source: Foods. 2026 Sep 21;15(18):3344. doi: 10.3390/foods15183344 (PMC13606302; doi:10.3390/foods15183344)
Supplement: Supplementary file 1 [file foods-15-03344-s001.zip › foods-4504780-supplementary.pdf]

Table S1 Digestion data of various samples

Time/min	NM-50	NM-80	50/50-50	50/50-80	HM-50	HM-80
0	0.7±1.0[a]	1.1±1.5[a]	0.5±0.7[a]	0.8±1.2[a]	1.5±2.1[a]	1.1±1.6[a]
5	25.0±1.5[b]	22.9±0.4[b]	23.8±0.1[b]	21.8±0.2[b]	15.7±1.3[a]	22.8±1.3[b]
10	47.5±0.2[d]	42.6±2.2[cd]	38.3±2.8[bc]	37.2±3.4[bc]	28.1±0.5[a]	33.6±0.3[ab]
15	60.6±0.2[c]	57.5±1.5[c]	51.3±2.1[b]	46.3±0.4[b]	31.1±1.4[a]	47.3±1.7[b]
20	67.1±0.2[d]	63.0±1.4[d]	53.4±4.3[c]	45.4±1.3[b]	36.9±1.5[a]	48.6±0.7[bc]
30	70.8±1.0[d]	73.4±0.1[d]	61.1±1.6[c]	51.2±1.3[b]	39.1±1.5[a]	54.2±0.1[b]
45	73.3±1.8[d]	77.1±1.6[d]	61.4±0.5[c]	50.9±0.1[b]	44.9±0.1[a]	57.4±0.4[c]
60	75.1±2.4[d]	76.4±1.9[d]	62.8±0.8[c]	52.7±0.2[b]	45.6±0.3[a]	58.0±1.0[bc]
90	75.3±0.9[d]	74.8±1.1[d]	63.2±0.5[c]	57.6±0.9[b]	48.4±1.6[a]	57.5±0.7[b]
120	76.3±2.5[c]	76.6±3.6[c]	64.0±1.2[b]	57.8±0.6[b]	47.9±0.8[a]	58.0±1.5[b]
180	78.7±0.9[d]	75.8±0.2[d]	68.0±1.0[c]	58.3±1.7[b]	51.7±0.8[a]	59.5±1.3[b]
240	82.7±1.1[d]	79.2±0.2[d]	68.0±0.8[c]	60.0±2.0[b]	51.7±0.8[a]	59.6±2.5[b]
300	81.7±0.3[d]	81.2±3.1[d]	72.6±1.9[c]	65.6±0.8[b]	51.3±0.3[a]	61.4±1.8[b]

Mean ± SD calculated from duplicate measurements. Values with different letters in the same row are significantly different with $p < 0.05$.
